# Potential Moderators of Physical Activity on Brain Health

**DOI:** 10.1155/2012/948981

**Published:** 2012-12-09

**Authors:** Regina L. Leckie, Andrea M. Weinstein, Jennifer C. Hodzic, Kirk I. Erickson

**Affiliations:** ^1^Department of Psychology, University of Pittsburgh, Sennott Square 3417, 210 S. Bouquet Street, Pittsburgh, PA 15260, USA; ^2^Center for the Neural Basis of Cognition, Department of Psychology, University of Pittsburgh, Sennott Square 3417, 210 S. Bouquet Street, Pittsburgh, PA 15213, USA

## Abstract

Age-related cognitive decline is linked to numerous molecular, structural, and functional changes in the brain. However, physical activity is a promising method of reducing unfavorable age-related changes. Physical activity exerts its effects on the brain through many molecular pathways, some of which are regulated by genetic variants in humans. In this paper, we highlight genes including apolipoprotein E (APOE), brain derived neurotrophic factor (BDNF), and catechol-O-methyltransferase (COMT) along with dietary omega-3 fatty acid, docosahexaenoic acid (DHA), as potential moderators of the effect of physical activity on brain health. There are a growing number of studies indicating that physical activity might mitigate the genetic risks for disease and brain dysfunction and that the combination of greater amounts of DHA intake with physical activity might promote better brain function than either treatment alone. Understanding whether genes or other lifestyles moderate the effects of physical activity on neurocognitive health is necessary for delineating the pathways by which brain health can be enhanced and for grasping the individual variation in the effectiveness of physical activity interventions on the brain and cognition. There is a need for future research to continue to assess the factors that moderate the effects of physical activity on neurocognitive function.

## 1. Introduction

One in every eight US seniors over the age of 65 has been diagnosed with Alzheimer's disease (AD), amounting to more than 5.4 million people. With the aging baby boomers, this number is predicted to double by 2050 [1]. Unfortunately, pharmaceuticals have had limited success in preventing or treating age-related cognitive dysfunction, such as AD or even normal cognitive aging. Fortunately, physical activity appears to be a promising nonpharmaceutical method to attenuate cognitive dysfunction in late life [2, 3]. Yet, there remain many unanswered questions about the effectiveness of physical activity to improve brain health, prevent dementia, and reduce age-related cognitive decline.

 “Physical activity” is an umbrella term defined by the Center for Disease Control (CDC) as any activity that increases heart rate and energy expenditure from one's basal level [4]. Examples of physical activities are walking, gardening, or even household chores such as cleaning. In turn, “exercise” is defined as a subcategory of physical activity, being any structured or repetitive activity that aims to improve fitness, endurance, or health such as strength training, purposefully running, or swimming. Both exercise and physical activity are often measured through self-report questionnaires; however, they can be assessed by identifying “fitness,” which is comprised of one's cardiorespiratory and skeletal muscle endurance, flexibility, and balance [4]. Fitness is objectively measured by an assessment of the maximum amount of oxygen consumption by carbon dioxide expulsion or VO_2_ max, as this is an objective measure of cardiorespiratory strength [5]. An individual who has a very low VO_2_ max or reports very low levels of physical activity or exercise is considered “inactive” or sometimes referred to as “sedentary,” but precise definitions of “sedentary” has become a matter of debate [6]. As a result, cross-sectional studies compare inactive or sedentary subjects to those with greater amounts of physical activity or higher fitness levels. In contrast, intervention studies usually recruit inactive individuals with the goal of improving fitness levels by increasing physical activity through engagement in exercise.

Numerous studies, reviews, and meta-analyses have documented that greater amounts of physical activity and higher fitness levels and are associated with greater gray matter volume [7–10], greater white matter integrity [8, 11–13], elevated functional dynamics including heightened connectivity of fronto-parieto-hippocampal circuits [14–17], and enhanced cognitive performance [12, 18]. Yet, despite the favorable effects of physical activity and cardiorespiratory fitness on brain health and cognitive function, there remains significant individual variability in the extent to which any individual benefits from physical activity. For example, randomized controlled trials of physical activity indicate that a subset of individuals often show little cognitive or neural benefit even after spending 12 months in an intervention [19]. On the other hand, others in the intervention show little improvement in cardiorespiratory fitness, while still demonstrating significant improvements in cognitive and brain function [20]. These results suggest that there might be factors moderating the effects of physical activity. Moderating factors could act by either attenuating or augmenting the effects of physical activity on neurocognitive function. For example, it is possible that if greater physical activity is accompanied by an increase in cognitive stimulation that the favorable effects of physical activity on cognitive and brain function could be magnified. On the other hand, if increased physical activity is accompanied by a poor diet, then the benefits of activity might be significantly attenuated. For this reason, it is important for studies to reflect on the factors that are contributing to variation in the effectiveness of physical activity to influence neurocognitive health. Indeed, instead of simply considering individual differences as noise in the analysis they could be considered as (1) an important source for understanding the effectiveness of interventions, (2) a method for identifying potential mechanisms by which physical activity exerts its effects on the brain, and (3) a way to tailor physical-activity-based treatments to more successfully enhance cognitive function for the greatest number of people. 

One of the challenges facing researchers in this field is that the list of possible moderating factors is virtually endless, making it difficult to identify which factors might be the most immediately relevant and the most important to study. However, there are several moderating factors that share characteristics with physical activity or have similar putative mechanisms or pathways underlying the effects that provide researchers with a theoretical platform to begin their investigation. Along these lines, pertinent moderators of the effects of physical activity on brain health include (1) genotypes, such as apolipoprotein E (APOE) [21], brain derived neurotrophic factor (BDNF) [22], and catechol-O-methyltransferase (COMT) [23] because these genes influence molecular pathways thought to be regulated by physical activity and (2) dietary variables, such as omega-3 fatty acids [24] that share molecular mechanisms and behavioral outcomes with physical activity. Although there are clearly other factors that are moderating the effects of physical activity on neural outcomes, we will focus this paper on these few moderators for which there is empirical support for their effects. Therefore, in this paper we first review current literature on physical activity and the aging brain (see Table 1 for select studies' characteristics) in an effort to reveal the brain circuits influenced by physical activity. Secondly, we focus on several factors that might moderate the effects of physical activity on brain health and use this evidence to recommend future research directions with the hope of more comprehensively understanding the factors that influence cognitive and brain aging.

## 2. Physical Activity and Gray Matter (GM**)** Volume

There is increasing evidence that the brain remains plastic throughout the lifespan and that a greater amount of physical activity or higher fitness levels take advantage of this natural characteristic of the brain. For example, individuals reporting greater amounts of physical activity or who are more physically fit have greater GM volume than individuals reporting less activity [7] or are less physically fit [39–41]. The effect of greater amounts of physical activity on GM volume can be found throughout the brain; however, the prefrontal cortex [8, 41–43] and hippocampus [9, 19, 40, 44] appear to be especially sensitive to these effects. The prefrontal cortex is associated with executive functions, a group of goal-oriented tasks that involve working memory, verbal fluency, and multitasking. The hippocampus, on the other hand, is involved in memory consolidation, spatial learning, and other memory processes. What makes the prefrontal cortex and hippocampus of particular interest is that these regions are some of the earliest regions to atrophy in late adulthood [45–48]. 

A technique called voxel-based morphometry (VBM) is one way to measure GM volume using high-resolution brain images from magnetic resonance imaging (MRI). This technique uses an automated segmentation algorithm to demarcate gray matter, white matter, and cerebrospinal fluid. Then, on a point-by-point basis throughout the brain the volume or density of gray matter can be examined as a function of any variable of interest (e.g., fitness levels). Studies using VBM have shown widespread age-related atrophy in frontal brain regions [45, 49, 50]. For example, Good et al. [49] examined GM volume in 465 adults as a function of age using VBM and demonstrated significant GM volume loss in several frontal and parietal regions. These results, along with many others, confirm the age sensitive nature of frontal GM volume and highlight the frontal lobe as an area of interest in aging research.

Fortunately, physical activity appears to increase GM volume in the frontal cortex [8]. In a 6-month intervention, older adults were randomly assigned to either a moderate intensity aerobic walking group or to a stretching and toning control group. The walking group was monitored by exercise coordinators and exercised 3 days per week. The control group received similar amounts of social contact with the exercise coordinators, but partook in stretching exercises instead of walking. After the 6-month intervention, the aerobic walking group showed an increase in gray matter volume in the frontal cortex, along the medial wall near the anterior cingulate cortex (ACC) and increased white matter volume in the genu of the corpus callosum, whereas the control group showed typical reductions in volume in these same regions. The results from this study indicate that only 6 months of moderate intensity physical activity is sufficient for increasing volume in brain areas that usually shrink during this period of life. 

The hippocampus, on the other hand, is involved in spatial navigation and memory. Located in the medial temporal lobe, the hippocampus is also prone to age-related atrophy [40, 46]. Important for memory formation and recall, hippocampal decay may be responsible for many of the symptoms associated with AD [19, 51], but even normal aging is associated with reduced hippocampal volume [52]. Rodent models of AD have shown decreased learning and memory performance accompanied by significant atrophy or even absence of hippocampal tissue [53–55]. Fortunately, the hippocampus is highly sensitive to exercise. In a study by van Praag et al. [56], mice that had access to a running wheel showed a significant increase in hippocampal cell proliferation and survival of new cells when compared to nonrunning mice. These and many other studies [57–59] indicate that exercise affects both the morphology and physiology of the hippocampus, including neurogenesis in the dentate gyrus. 

Participation in moderate intensity exercise also appears to affect the hippocampus in humans. In an intervention extending for 1 year, participants were randomly assigned to either a moderate intensity aerobic walking group or to a stretching and toning control group, similar to the 6-month study described above [19]. Over the year, the control group experienced normal age-related atrophy of the hippocampus. However, the walking group experienced a 2% increase in hippocampal volume over this same period. In addition, hippocampal atrophy rates within the control group were inversely related to preintervention fitness levels, indicating that higher fitness levels were protective against hippocampal atrophy. This study provides some key insights into the effects of exercise on the hippocampus: first, only moderate intensity physical activity for 1 year is sufficient for increasing the volume of the hippocampus during a time period of life when the hippocampus tends to decline in size and increasing the risk for dementia. Second, higher fitness levels could reduce the rate of hippocampal decay over a 1 year period. While this study was conducted using healthy adults, these results identify aerobic exercise as a potentially effective method for preventing and treating diseases, such as Alzheimer's disease and dementia, that are associated with hippocampal atrophy.

Recent evidence has also linked the associations between higher fitness levels and greater GM volume with improvements in cognitive function. For example, Erickson et al. [40] found that higher cardiorespiratory fitness levels were associated with increased hippocampal volumes, which in turn mediated the relationship between fitness and spatial memory performance. A similar relationship was found between higher fitness levels, greater prefrontal cortex volume, and better performance on measures of executive control [41]. Thus, increased GM resulting from exercise and associated with higher fitness levels appears to have a significant association with improved cognitive function. 

## 3. Genetic Moderators of Physical Activity ****on Brain Health

Many different factors may moderate the effect of physical activity on brain health. By including these variables as statistical interaction terms (i.e., physical activity × genotype), the effect of moderation on cognitive or neural outcomes can be quantified. In this way, genetic variants such as APOE, BDNF, and COMT may explain some of the individual differences in the extent to which physical activity augments brain health [60]. Effect sizes in these studies are often difficult to directly compare mainly because of the variation in samples, designs, and candidate genes studied. The genes in this paper have been chosen based on animal literature as well as some supporting human research.

### 3.1. Apolipoprotein E (APOE)

The APOE gene has three different allele variations: *ε*2, *ε*3, and *ε*4. The majority of the population carries the *ε*3 allele, whereas the *ε*2 and *ε*4 alleles are less common [61]. The gene APOE creates the lipoprotein of the same name, abbreviated ApoE. ApoE is found throughout the body and is linked to cardiovascular functions such as the transport of fats and cholesterol from the blood stream to the liver for processing. There have now been numerous studies linking the APOE gene to cognitive performance and risk for dementia [62, 63]. In particular, the APOE *ε*4 allele is considered a potent risk factor for AD [64]. The APOE *ε*4 variant also influences cognition in healthy adults without dementia. For example, Reinvang et al. [30] examined whether memory and executive functioning varied by APOE genotype in a group of healthy adults. APOE *ε*4 carriers performed more poorly on letter and digit span tasks as well as the Stroop color-word task than noncarriers. 

**β**-amyloid (A**β**) plaques are also biomarkers and putative causes of AD and are closely linked to APOE genotype [65–68]. The ApoE protein produced by APOE *ε*4 can bind to A**β** more efficiently than the other allele variants, increasing the number of plaques, which are thought to lead to AD symptoms [65]. Rodent studies reveal APOE *ε*4 knockout mice have reduced A**β**deposition [66], further confirming APOE genotype as a risk factor for impaired clearance of A**β**and susceptibility for AD. While A**β** plaque deposition is a putative cause of AD, voluntary physical activity effectively reduces amyloid plaque deposits in rodents [27, 28, 69, 70]. Exercise may have this effect by promoting the expression of growth factors that are associated with reduced amyloid and changes in inflammatory pathways that alter A**β** plaque deposition. For example, a study by Parachikova et al. [28] used transgenic (Tg2576) mice as a model for AD where the particular mutation produces an abundance of amyloid precursor protein (APP), a protein that gets cleaved to produce amyloid. The results revealed a significant decrease in mRNA for inflammatory biomarkers in Tg2576 mice with 3 weeks access to a running wheel when compared to Tg2576 mice without access to running wheels. Nichol et al. [27] extended this to show that reduced mRNA for inflammatory biomarkers coincided with decreased A**β** plaque deposition. Whether participation in physical activity moderates plaque deposition in humans has only recently been studied. 

Consistent with the rodent literature, several epidemiological studies have also reported that the association between greater amounts of physical activity and reduced risk of dementia might be dependent on APOE genotype. For example, Podewils et al. [29] examined 3,375 individuals in the United States, measuring physical activity levels with a modified Minnesota Leisure Time Activity Questionnaire. From this measure the authors categorized the activities into groups based on metabolic equivalents, creating a calculation of energy expenditure for each individual. Adults aged 65 and over who engaged in regular physical activity had a markedly reduced risk of developing AD after a 5-year follow-up than those who were sedentary. Interestingly, these results were enhanced in APOE *ε*4 noncarriers, such that *ε*4 carriers showed no significant changes in risk associated with increased participation in physical activity. Although one strength of this study is its large sample size, the self-reported nature of physical activity along with its observational design make it difficult to conclude the direction and strength of the association. 

In contrast, several other studies have found that APOE *ε*4 carriers benefit more from exercise than do non-*ε*4 carriers. Schuit et al. [21] examined the relationship between levels of physical activity and cognitive decline in late adulthood as a function of the presence or absence of the *ε*4 allele in 347 elderly Dutch men. The infrequent exercising group, defined as those being physically active less than one hour per day, had a twofold increased risk of cognitive decline over the three-year period as measured by the Mini Mental State Exam, but this risk was mitigated in the more frequent exercising APOE *ε*4 carriers. Hence, this epidemiological study suggests that participation in regular exercise could offset genetic risk factors for dementia. Consistent with this finding, a study of nondemented middle-aged adults found that greater amounts of physical activity was associated with faster performance on a memory task and greater brain activation in APOE *ε*4 carriers compared to their more sedentary APOE *ε*4 peers [71]. Again, although provocative, the results from these studies need to be interpreted within the context that physical activity was assessed using self-reported measures, and these studies were observational in nature and not stemming from randomized controlled trials.

Evidence in AD patients shows a slightly different pattern than that described above. For instance, Honea et al. [25] examined whether the association between fitness and brain volume in 61 individuals with early-stage AD would be moderated by the APOE polymorphism. Consistent with previous findings of a cross-sectional study examining VO_2_ peak in dementia and early AD patients [10], higher aerobic fitness levels (VO_2_ peak) were related to greater brain volume in the medial temporal lobe in early AD patients, as measured using voxel-based morphometry. Surprisingly, APOE *ε*4 status did not moderate the relationship between fitness and brain volume in either the AD patients or in the 56 healthy control participants. Contrary to this finding, Smith et al. [15] examined the effect of physical activity and APOE *ε*4 on brain activity during memory processing in older (65–85) cognitively intact adults. They found that physical activity increased semantic memory-related brain activation in cognitively intact older adults who carry the APOE *ε*4 allele. However, the authors included only 17 subjects per group and assessed physical activity through the self-report Stanford Brief Activity Survey (SBAS), rather than an objective measure such as VO_2_ peak. Finally, in a study of amyloid deposition in older adults (55–88), greater amounts of self-reported physical activity were associated with reduced plaque deposition in APOE *ε*4 carriers [26]. Hence, despite some variability in the literature, there is a growing consensus that the harmful effects of the APOE *ε*4 allele are mitigated by greater amounts of physical activity. Yet, despite this preliminary conclusion, there is a need for more research to determine if APOE *ε*4 carriers who were randomly assigned to receive exercise could benefit more from an intervention than their non-*ε*4 counterparts. There is also a great need for larger sample sizes, as the costly nature of neuroimaging has limited the number of participants assessed in previous studies.

### 3.2. Brain-Derived Neurotrophic Factor (BDNF)

BDNF is another gene that could be moderating the effectiveness of physical activity on brain health. Similar to APOE, BDNF refers to both the gene and its resulting protein. There is a valine to methionine substitution in the BDNF gene at codon 66 resulting in several natural occurring variants of this gene in the human population. The Val/Met and Met/Met genotypes have been associated with decreased cognitive performance [72–74] and GM volume [75, 76] compared to Val homozygotes. The Met allele is associated with a reduction in the regulated secretion and trafficking of BDNF from the cell, which is thought to impact the role of BDNF in cell proliferation and learning.

While present in all brain areas, BDNF is highly concentrated in the hippocampus and cortex [77], promoting cell proliferation and signaling through several pathways, most notably by binding to the receptor tyrosine kinase B (TrkB). BDNF binding to the TrkB receptor depolarizes glutamatergic cells and results in several intracellular signaling cascades that moderate mRNA transcription [78, 79]. 

Additionally, TrkB binding phosphorylates the NMDA receptors of the postsynaptic neuron, increasing cyclic-AMP production and promoting long-term potentiation (LTP) [80]. In the hippocampus, LTP is considered a cellular analog of learning and is closely coupled with behavioral indices of learning and memory [81]. Rodent models where BDNF-receptor binding has been blocked or in BDNF knockouts, animals show deficits in acquisition and retrieval on the Morris water maze [82]. Mu et al. [32] observed a decrease in spatial memory retention in rats treated with a BDNF blocking antibody, providing further evidence that BDNF is necessary for the facilitation of learning and memory. These studies suggest that BDNF binding is critical to several cognitive processes. 

Interestingly for this discussion, BDNF levels also increase as a function of both immediate [83] and longer-term [33] exercise. In fact, increased BDNF expression is a highly replicated effect in exercise studies [84]. For example, Stranahan et al. [33] examined amounts of voluntary wheel running in mice, finding that hippocampal BDNF levels increased with greater amounts of exercise, and in turn, higher levels of hippocampal BDNF was associated with greater dendritic spine density and hippocampal neurogenesis. Thus, increased BDNF signaling is considered one of the primary molecular pathways by which exercise improves neurocognitive function.

BDNF is also found outside of the central nervous system and can be measured in serum and plasma in humans. While it has been argued that serum levels are not a valid measure of cortical BDNF levels [85], rodent studies have found strong correlations between hippocampal and frontal BDNF levels and peripheral BDNF measures [86]. Human studies report that BDNF levels are a reliable biomarker for depression, where there is a strong negative relationship between peripheral BDNF levels and depression [87] and treatment of depression might result in an increase in serum BDNF [88]. Postmortem studies of individuals who have died from suicide also report that cortical levels of BDNF closely correspond to serum levels in individuals diagnosed with depression [89]. Other studies of serum or plasma BDNF find that levels are correlated with measures of brain integrity including hippocampal volume [31] and cognitive function [90]. Yet, despite these correlations, the translation from peripheral to cortical BDNF levels in humans is still unknown as is the significance of BDNF in the serum. Nonetheless, measuring BDNF in the serum allows for a minimally invasive proxy for examining a biomarker of brain health that is simply mirroring what is happening in the brain. From this perspective, given that animal studies have consistently reported increased BDNF levels after exercise, it has become a molecule of interest in human studies of physical activity and exercise. In fact, several studies have reported that acute bouts of physical activity are effective at increasing BDNF levels in serum [85]. In humans, Yarrow et al. [91] examined BDNF by sampling serum levels 0, 30, and 60 minutes into a physical activity session, where participants rode a stationary bike at a moderate pace, as well as 30 minutes after a brief cooldown. They demonstrated that BNDF levels were significantly elevated during and immediately after the physical activity session. Long-term exercise interventions, however, have not shown elevated levels of serum BDNF at follow-up [19] indicating some differences between the effects of acute and long-term exercise on BDNF pathways. The differences between acute versus long-term effects of exercise on BDNF levels remains a matter of speculation.

BDNF clearly remains one of the primary molecular pathways by which exercise is thought to influence cognitive function. In line with this hypothesis, hippocampal volume is correlated with serum BDNF levels in older adults [31], and exercise-induced increases in hippocampal volume are associated with increased BDNF serum levels [19]. Although there is not a one-to-one association between BDNF protein and the BDNF polymorphism, the link between the Met allele and reduced secretion and trafficking of the protein suggests that physical activity could play an important moderating role. Unfortunately, to date, there have not been published investigations of whether physical activity moderates the effect of the BDNF polymorphism on neurocognitive function. This will be an important avenue for future research. 

### 3.3. Catechol-O-methyltransferase (COMT)

COMT is a gene that produces an enzyme of the same name and has been hypothesized to influence cognitive function and brain health. The COMT enzyme works by breaking down the neurotransmitters dopamine, epinephrine, and norepinephrine. COMT is located mainly in the frontal cortex and is necessary for the regulation of the aforementioned neurotransmitters but is predominantly associated with dopamine [92]. Dopamine is concentrated in the prefrontal cortex, where it plays a role in executive control [93, 94]. The COMT enzyme is altered by its genotype where a valine (Val) to methionine (Met) amino acid substitution at the 158/108 locus of the peptide sequence affects the thermostability of the enzyme. The COMT gene therefore has three variants, Val/Val, Val/Met, and Met/Met, where Val carriers show increased enzyme activity, which in turn reduces dopamine levels at a faster rate in the synapse [92]. Wishart et al. [35] found that Val homozygotes displayed decreased performance on executive tasks. Additionally, de Frias et al. [34] revealed reduced cognitive performance and greater cognitive decline after a 5-year follow-up in older adult Val/Val individuals. There have now been numerous studies documenting the association between the COMT gene and cognitive performance; yet several recent reviews and meta-analyses have questioned the robustness and consistency of the effect across populations and tasks [95]. Such variation suggests that there might be factors that are moderating the effect of the COMT gene and influencing the direction and consistency of the effect. 

One such moderator might be participation in physical activity. Physical activity influences dopaminergic circuitry in both the prefrontal cortex and basal ganglia in animal models [96–99] suggesting that its effects on cognitive function might be partially mediated by its effects on dopamine. Unfortunately, few studies have examined whether the COMT gene moderates the effect of physical activity on neurocognitive function. In one study, Stroth et al. [23] examined whether variants of the COMT gene would be moderated by participation in a 17-week physical activity intervention in a sample of 75 adults. At the end of the intervention, Val carriers who were in the running group displayed disproportionately faster response times on a working memory paradigm when compared to Met homozygotes, indicating that the benefits associated with physical activity on working memory function were dependent on the COMT genotype. This effect could be happening through the effects of physical activity on the gene itself, through physical activity effects on dopamine, or on dopamine receptors. In other words, the pathways by which this effect occurs remain a matter of speculation. 

The evidence outlined above marks the COMT gene as a possible moderator of the effects of physical activity on cognitive function, but more studies are needed to replicate this effect and expand it to larger sample sizes and other populations. However, there is a striking resemblance between the moderating effects of physical activity on APOE and COMT genotypes. In each instance there is speculation or evidence that participation in physical activity might mitigate the effect of the gene on cognitive function. 

## 4. Dietary Moderators of Physical Activity**** on Brain Health

Omega-3 fatty acids provide many beneficial effects to the body [100] and brain [101], and the pathways by which omega-3 fatty acids work on the body are similar to the pathways thought to be regulated by physical activity. Hence, several human and animal studies have speculated about the additive or multiplicative benefits that might arise from combining omega-3 administration or supplementation with greater amounts of physical activity [102]. 

There are two distinct types of omega-3 fatty acids: docosahexaenoic acid (DHA) and eicosapentaenoic acid (EPA). DHA is primarily located in the cortex and is linked to a variety of cognitive functions such as attention and memory. EPA, however, is found all over the body and is known for its anti-inflammatory effects on the cardiovascular system. Higher DHA levels in the serum and self-reported dietary intake of DHA have been associated with better performance on measures of executive function [103, 104]; however many of these studies have been either cross sectional in nature or consisting of rather small sample sizes. On a molecular level, DHA is critical to cell membrane structure, fluidity, and ion permeability and is synthesized in small amounts in humans [105, 106]. As a result, humans are required to consume most of their necessary DHA to maintain appropriate levels. 

DHA has also been linked to dopamanergic pathways via the D2 dopamine receptor [107–109]. Rodents consuming a DHA deficient diet show significantly fewer D2 receptors in the striatum, mimicking similar pathology to rodent models of depression [110]. Rodent models of Parkinson's disease, where dopamanergic cells and receptors are significantly reduced, demonstrate that DHA supplementation is associated with a greater number of dopamanergic cells in the substantia nigra when compared to mice consuming a DHA deficient diet [111]. DHA is also under investigation as a potential nonpharmaceutical treatment for schizophrenia because of its effect on maintenance and preservation of normal dopaminergic function [112]. 

While both EPA and DHA are currently being explored as nonpharmaceutical treatments for schizophrenia, Parkinsons' disease, and other psychiatric and neurological conditions, other research is examining its links to cognitive function in middle-aged and geriatric populations. Muldoon et al. [36] tested the association between DHA serum levels and measures of cognitive function in midlife adults. They recruited 280 volunteers between 35 and 54 years of age, free of major neuropsychiatric disorders, who were not taking fish oil supplements. Individuals with higher DHA levels performed better on tasks of nonverbal reasoning and mental flexibility, working memory, and vocabulary. They concluded that DHA is related to cognitive health throughout the lifespan and may influence the prevalence of neuropsychiatric and impaired cognitive function in late life. However, this study was cross sectional, as are many others on DHA, so the directionality of the effects remains questionable.

In fact, some evidence suggests DHA might play an important role in dementia and age-related cognitive decline [113]. Individuals with self-reported diets high in omega-3 fatty acids decline at a slower rate on the MMSE as compared to omega-3 deficient individuals [38]. Several reviews of the literature and epidemiological studies over the past 10–12 years have revealed differing results, however, when conducting randomized trials of DHA supplementation or longitudinal studies following individuals with variation in fatty acid intake [114]. There is some evidence that greater intake of DHA might help to prevent or delay AD by preventing tangles and A**β** plaques [37, 115]. Although some research has hypothesized that DHA may be effective at reducing A**β** plaques, no consistent evidence for the treatment of AD has been found in association with DHA supplementation. Jicha and Markesbery [116] outline both epidemiological and clinical studies and conclude that there is evidence that DHA supplementation may act in cellular preservation but cannot be considered a reliable treatment for AD. 

Physical activity, however, may provide an avenue by which the effects of DHA on cellular integrity and cognitive function may be enhanced [102, 106]. Chytrova et al. [24] revealed an additive effect of physical activity and DHA supplementation in a rodent model of synaptic plasticity and membrane structure in the dentate gyrus of the hippocampus. Mice in the DHA condition showed increased levels of membrane-bound synaptic proteins when compared to mice in the regular diet condition; however those mice receiving both DHA supplementation and physical activity showed greater levels of synaptic proteins than their counterparts not receiving physical activity. These results suggest that physical activity potentiated the effects of DHA supplementation on membrane proteins associated with synaptic plasticity. Unfortunately, studies in humans have not yet determined whether the effects of DHA on cognitive function are moderated by participation in physical activity. Given the molecular and cellular associations described above, it would be reasonable to hypothesize that such an interaction might be present.

## 5. Conclusion

Physical activity is effective at enhancing cognitive and brain function; however, many genetic and behavioral factors may be attenuating or augmenting the effect of physical activity. Genes such as APOE, BDNF, and COMT share molecular pathways with exercise and therefore might have the potential to moderate the effect of physical activity on cognitive function and neuronal health. For example, DHA reduces A**β** plaque build-up, similar to APOE, functions in the cell membrane, targets the synapse, similar to BDNF, and has strong ties to the dopaminergic pathway similar to COMT. Physical activity also affects all of these pathways, further weaving these independent variables together as part of a much larger story. 

Unfortunately, many studies only assess one or two variables at a time in relation to cognitive function or physical activity. Therefore, the influence of genes or dietary variables on the effect of physical activity remains largely speculative. In fact, in this paper we highlight the evidence that there might be interactions between these variables, but to date, few studies have formally assessed these associations. Future studies with larger sample sizes and more comprehensive assessments of these variables are needed to move this field forward. Individual variability in prior studies highlights the need to more closely examine the potential moderators described here. 

In addition, the way in which each of the genetic variables moderates the effect of physical activity is largely unknown. While there is evidence for physical activity to increase mRNA transcription of BDNF and COMT, the exact promoters targeted, and the way in which they are activated has not been identified. Animal models could provide insight to the pathways involved in the moderating effect of certain genotypes on physical activity and are a critical starting point for understanding these potential moderators of physical activity on neuronal changes, brain health, and cognitive function.

It will be important for new studies to take into consideration the individual variability contributed by dietary variables such as omega-3 levels and genes. These variables are clearly a source of “noise” within the data and could contribute to explaining the effects, or lack thereof, of physical activity on cognitive function. As we stated earlier, it will be important for future research to consider these variables as potential moderators instead of just “noise.” 

In sum, prior reviews and meta-analyses have focused on the protective and enhancing effects of exercise on neurocognitive function [12]. In this paper we have taken a different approach and have described several potential moderating factors that could be contributing to variation in the effectiveness of physical activity to enhance cognition. Although there is a long list of potential moderators, we have chosen here to focus on three different genes and a single dietary factor. This of course does not preclude other potentially important moderators such as intellectual engagement, other genes, other dietary factors, age, sex, or the combination of different types of physical activities (e.g., resistance training paired with aerobic training). We argue that it will be important for future research to consider these variables when designing studies, analyzing data, and interpreting the results from physical activity studies.

## Figures and Tables

**Table 1:** Characteristics of selecting reviewed studies.

First author (citation no.)	Experimental method	Measure(s)	Population mean age (standard deviation)	*N *	Significant finding(s)
APOE as variable of interest					

Honea et al. [25]	Cross sectional: APOE *ε*4 carrier status, dementia status × global connectivity, GM volume, cognitive performance, and fitness Nondemented (*n* = 56) Early-stage AD (*n* = 61)	APOE *ε*4 carrier status VO_2_ peak WMS III WAIS MMSE Voxel-based morphometry Diffusion tensor imaging	Older adults aged 73.4 (6.3)	117	Higher aerobic fitness levels were related to greater brain volume in the medial temporal lobe in early AD patients.
Liang et al. [26]	Cross sectional: APOE *ε*4 carrier status × fitness, biomarker risk status	Walking, running, and jogging questionnaire PIB APOE *ε*4 carrier status	Healthy older adults aged 55–88	56	Physical activity was associated with reduced plaque deposition in APOE *ε*4 carriers.
Nichol et al. [27]	Control 1: nontransgenic mice Control 2: Tg2576 mice Experimental group 1: nontransgenic mice with 3 weeks access to running wheel Experimental group 2: Tg2576 mice with 3 weeks access to running wheel	IL-1*β* TNA-*α* A*β*	Tg2576 transgenic mice (*n* = 29) C57Bl6/SJL nontransgenic mice (*n* = 27)	56	IL-*β* and A*β* fibrils are significantly lower in transgenic mice with access to running wheel than transgenic control mice.
Parachikova et al. [28]	Control 1: nontransgenic mice (*n* = 6) Control 2: Tg2576 mice (*n* = 6) Experimental group 1: nontransgenic mice with 3 weeks access to running wheel (*n* = 6) Experimental group 2: Tg2576 mice with 3 weeks access to running wheel (*n* = 6)	Morris water maze latency A*β* mRNA expression CXCL1 and CXCL12 protein	Tg2576 transgenic mice C57Bl6/SJL nontransgenic mice	24	Transgenic mice with voluntary exercise performed significantly better on Morris water maze than control transgenic mice showed no increase in inflammatory markers and increased mRNA production of CXCL1.
Podewils et al. [29]	Cross sectional: APOE *ε*4 carrier status × onset of dementia × physical activity	Minnesota leisure time activity questionnaire APOE *ε*4 carrier status	Adults aged >65	3,375	Adults who engaged in regular physical activity had significantly reduced risk of developing AD after a 5-year follow-up than those who were sedentary. *ε*4 carriers showed no significant changes in risk associated with increased participation in physical activity.
Reinvang et al. [30]	Cross sectional: APOE *ε*4 (*N* = 69) carriers versus non-*ε*4 carriers (*N* = 117)	AX-Continuous Performance Task WAIS II Color-Word Interference Task CVLT-II	Healthy adults mean age 64.5	186	There is a negative relationship between *ε*4 carrier status and performance on working memory tasks where *ε*4 carriers performed worse than noncarriers.
Schuit et al. [21]	Cross sectional: activity status × APOE *ε*4 carrier status, cognition Inactive group: self-report less than 1 hr/day physical activity Active group: more than 1 hr/day physical activity	MMSE APOE *ε*4 allele carrier status	Healthy older adult males 74.6 (4.3)	347	Inactive group showed significant decrease in MMSE score over 3 years. APOE *ε*4 carriers showed significantly steeper decline than *ε*4 noncarriers.
Smith et al. [15]	Cross sectional: APOE *ε*4 carrier status × fitness, cognitive function, fMRI connectivity, and grey matter volume	APOE *ε*4 carrier status Stanford Brief Activity Survey MMSE Mattis Dementia Rating Scale 2 Rey auditory verbal learning test Geriatric depression scale Whole-brain event-related fMRI Voxel-based morphometry	Healthy older adults aged 65–85	68	Effects of PA on task-related activation were reliably more pronounced in APOE-*ε*4 carriers with no significant differences in grey matter volume.

BDNF as variable of interest					

Erickson et al. [31]	Cross sectional: BDNF serum levels × grey matter volume, spatial memory performance	BDNF serum levels MRI first segmentation Spatial memory task	Healthy older adults mean age 66.5	142	BDNF levels mediated the relationship between the significant decline in hippocampal volume as a function of age.
Mu et al. [32]	Experimental group: rats treated with BDNF antibody (*n* = 7) Control group: IgG treated rats (*n* = 6)	Morris water maze latency Place navigation test Spatial probe test	Male Sprague-Dawley rats	13	BDNF antibody treated rats had longer learning latencies and spatial memory performance.
Neeper et al. [22]	Cross sectional: 0, 2, 4, or 7 days of running wheel access	BDNF mRNA levels in cortical regions	Sprague-Dawley rats	39	2, 4 and 7 days of exercise significantly increased BDNF mRNA levels in the hippocampus and caudate.
Stranahan et al. [33]	Control 1: nontransgenic mice (*n* = 12) Control group 2: nontransgenic mice with access to running wheel (*n* = 12) Experimental group 1: leptin receptor mutant (db/db) transgenic mice (*n* = 12) Experimental group 2: leptin receptor mutant (db/db) transgenic mice with access to running wheel (*n* = 12)	Open field activity Hippocampal BDNF protein levels	Male db/db transgenic mice Male C57Bl6/SJL nontransgenic mice	48	Access to a running wheel increased hippocampal BDNF levels in both wild type and transgenic mice.

COMT as variable of interest					

de Frias et al. [34]	Longitudinal: COMT genotype (Met/Met (*n* = 26); Val/Met (*n* = 46); Val/Val (*n* = 23)) × cognitive performance over 5-year period with 2 time points (0 and 5 years)	COMT genotype Verbal fluency Working memory task Tower of Hanoi task WAIS block design task	Healthy adult males aged 58.1 (12.86)	292	At baseline, COMT Met/Met group performed better than the Val/Val and Val/Met variants on executive function tasks and block design. Greater executive function decline after a 5-year follow-up in Val/Val individuals.
Stroth et al. [23]	Control: nonintervention (Met *n* = 19; Val *n* = 9) participants Experimental group: group running session 3x per week for 4 months (Met *n* = 33; Val *n* = 14)	2-back working memory task Stroop task Dots-mixed task Maximal field track fitness test COMT genotype	Healthy adults aged 22.7 (5.7)	75	Val/Val individuals in running intervention showed increased cognitive performance compared to Met carriers.
Wishart et al. [35]	Cross sectional: COMT genotype (Met/Met (*n* = 26); Val/Met (*n* = 46); Val/Val (*n* = 23)) × cognitive performance	COMT genotype Trail making task	Healthy adults aged 18–85	95	COMT Met/Met participants showed increased performance on trails B task than Val/Val or Val/Met genotypes.

Omega-3 fatty acid as variable of interest					

Chytrova et al. [24]	Control: regular diet, no access to running wheel Experimental group 1: regular diet, access to running wheel Experimental group 2: DHA-enriched diet, no access to running wheel Experimental group 3: DHA-enriched diet, access to running wheel	Morris water maze latency Hippocampal levels of STX-3, STX-1, NR2B, and GAP-43	Male Sprague-Dawley rats	24	Both DHA and access to running wheel increased levels of NR2B, STX-3, and Morris water maze learning.
Muldoon et al. [36]	Cross sectional: serum omega-3 fatty acid levels × cognitive performance	Serum omega-3 levels WMS-III WASI Stroop task	Healthy middle aged adults aged 44.6 (6.7)	280	Higher levels of DHA correlated to increased performance on several cognitive measures, specifically working memory.
Oksman et al. [37]	Control: standard diet Experimental group 1: high omega-6 diet Experimental group 2: high omega-3 diet	Morris water maze latency A*β*42 levels and plaques	Female F3 or F4 generation Transgenic mice (APPswe/PS1dE9)	21	Omega-3 enriched diet decreased A*β*42 levels compared to the two control diets.
van Gelder et al. [38]	Longitudinal: omega-3 fatty acid levels × cognitive function over 5 year period with 2 time points (0 and 5 years)	Dietary questionnaire MMSE	Healthy older Dutch males aged 70–89	228	Increased omega-3 fatty acid consumption was associated with smaller decline in cognitive function at 5-year followup.

Citation, experimental procedures, subject information, sample size, and main significant findings, are outlined for studies that have been highlighted as exemplary in text.

Weschler Memory Scale III (WMSIII), Weschler Abbreviated Scale of Intelligence (WASI), California Verbal Learning Test II (CVLTII), and Pittsburgh Compound B (PIB).
